# Improvement of Groin Pain in a Football Player with Femoroacetabular Impingement via a Correction of the Pelvic Position—A Case Report

**DOI:** 10.3390/jcm12237443

**Published:** 2023-11-30

**Authors:** Oliver Ludwig, Günther Schneider, Jens Kelm

**Affiliations:** 1 Department of Sport Science, Rheinland-Pfälzische Technische Universität Kaiserslautern, 67663 Kaiserslautern, Germany; 2 Clinic for Diagnostic and Interventional Radiology, Saarland University Medical Center Homburg, 66421 Homburg, Germany; dr.guenther.schneider@uks.eu; 3 Chirurgisch-Orthopädisches Zentrum Illingen, 66557 Illingen, Germany; jk66421@hotmail.de

**Keywords:** groin pain, cam impingement, football, soccer, muscular imbalance, femoroacetabular impingement, pelvic posture, pelvic tilt, hyperlordosis, kicking technique

## Abstract

*Background:* Femoroacetabular impingement is one possible cause for groin pain and can lead to long periods of absence for football players. In cam impingement, the end-grade position of the leg at kicking makes the hip particularly prone to faulty contact between the acetabulum and the femoral head. Studies suggest that the resting position of the pelvis in the sagittal plane may have an important role in the biomechanics of movement in the presence of cam impingement. *Methods*: A 19-year-old male competitive footballer complained of sudden groin pain during a period of low athletic load. Biomechanical tests (3D posture and isometric strength analyses) showed that unbalanced individual strength training had resulted in an increased forward tilt of the pelvis. At the same time, cam impingement was confirmed radiologically, which obviously contributed to the sudden onset of the symptoms. The kicking technique of the athlete showed increased hip and trunk flexion, which also indicated a muscular imbalance. Targeted strength and stretching exercises three times a week improved the pelvic position in terms of reduced anteversion. At the same time, the patient performed strength exercises to improve his kicking technique. *Results:* After 8 weeks, improvements in his pelvic position and global posture and increased muscle strength could be verified. At the same time, the athlete was free of complaints again. *Conclusions*: When groin pain occurs in football players with cam impingement, special attention should be paid to the resting position of the pelvis in the sagittal plane. Correcting increased pelvic anteversion can prevent unfavourable end-grade collisions of the acetabulum and femoral head during kicking with strong hip flexion and adduction. Possible changes in the pelvic position due to adverse individual strength training performed by young athletes should always be kept in mind.

## 1. Introduction

Groin pain in football players is a common phenomenon. A consensus statement has specified several entities that may be responsible for these symptoms [1]. Pathologies of the hip are identified as a possible cause of groin pain in athletes, with hip impingement playing an important role. Symptoms due to femoroacetabular impingement (FAI) occur frequently in football players [2,3], sometimes leading to long periods of sports absence [4], and this seems to increase the risk of other pathologies such as hamstring injuries [5]. A cam deformity of the hip is characterized by a non-spherical deformation of the anterolateral head–neck junction, which is forced into the acetabulum during flexion and internal rotation of the hip joint. Sports such as football are characterized by precisely these movement patterns when kicking. Over time and with repeated kicking movements, the CAM-FAI deformity can damage the soft tissue structures of the hip. CAM-FAI can lead to sudden pain, especially during kicking movements and rapid changes of direction, such as during movement feints in football [6].

The biomechanical conditions, especially the positioning of the head of the femur in relation to the acetabulum and their dynamic interplay during movement, are important factors that determine whether a morphological CAM-FAI will also become pathologically relevant [7]. Current biomechanical simulations show that there are correlations between CAM-FAI and biomechanical factors that can affect the acetabulum–femoral head contact, such as the pelvic tilt [8]. Thus, reducing an increased anterior pelvic tilt could influence the end-grade contact between the acetabulum and labrum and improve pain-free hip range of motion (ROM). Kobayashi et al. [9] simulated the influence of different pelvic tilt angles on hip ROM using pre- and postoperative computed tomography (CT) images of patients with CAM-FAI. They were able to show that a pelvic tilt reduction of 10° resulted in an improved ROM, with an effect similar to cam resection surgery. Patel et al. found that an increased anterior pelvic tilt led to an earlier onset of FAI and concluded that “rehabilitation aimed at altering the tilt of the pelvis may reduce the frequency of impingement and limit further joint damage” [10].

Before any surgical intervention, all conservative measures should be exhausted in CAM-FAI. Conventional physiotherapy measures for cam impingement are primarily aimed at strengthening the hip adductors or abductors, and the lower trunk as well as improving stability in a single-leg stance, depending on the patient’s individual deficits [11]. These therapy programs are generally carried out on a side-specific basis, whereby one-sided muscular imbalances (for example, between hip adduction and abduction) are to be compensated for.

On the other hand, arthroscopic recontouring of the femoral head–neck junction is considered the treatment of choice in adults after unsuccessful conservative treatment of CAM-FAI. A meta-analysis by Gatz et al. [12] recently concluded that arthroscopic hip surgery achieved better outcome values than a conservative physiotherapy-based treatment regime and must therefore be regarded as a more effective therapeutic treatment.

In view of the long absence times after cam impingement surgery, usually longer than 6 months in young footballers until their return to sports [13], an attempt to achieve pain relief by means of physiotherapeutic measures is reasonable, especially in high-performance sports. As mentioned above, the spectrum of conservative therapy is considered to be small; therefore, postural and movement analyses can be helpful to show biomechanical deviations and include correction of these in the therapy planning [14]. This is where this case study comes in.

Based on these considerations, the aim of our case study was to show whether improving the muscular balance of the hip muscles can improve the pelvic position, which in turn helps to avoid unfavourable acetabular–femoral head contact during athletic movements and ultimately leads to the symptoms subsiding. Instead of one-sided exercise programs, we performed symmetrical exercises to compensate for a dorso-ventral muscular imbalance with the aim of reducing the pelvic forward tilt and thus improving the biomechanical basis of hip movement under the condition of CAM-FAI. To the best of our knowledge, this has not yet been reported in this form in the literature.

## 2. Case Description

We report the case of a 19-year-old male competitive footballer (168 cm, 65 kg, BMI 23 kg/m^2^, body fat 11%, muscle mass 35 kg). The patient began playing football at the age of 7 years and is an attacking midfielder at a youth academy in the fourth highest division in Germany, with four regular training sessions per week and one match. In the middle of the season, the athlete complained of a sudden onset of severe left groin pain upon taking shots and changing direction, rated as 8–9 on a 10-level pain scale, without any preceding traumatic event. The dull pain in the left groin region and a feeling of stiffness in the left hip joint lasted for several hours after training and subsided overnight. The remarkable aspect was that the pain occurred for the first time during a period of low training and playing load for the athlete and therefore could not be attributed to an increased sporting load.

### 2.1. Medical Examination

A manual examination revealed no groin or symphysis tenderness, but a positive FADIR test (pain 8 out of 10 on flexion, adduction and internal rotation of the left hip joint) [10], which reproduced the pain complaint. The extent of internal rotation of the left hip joint was limited (Table 1). The apprehension test with extension, abduction and external rotation of the hip joint was slightly painful (4 out of 10). The proximal insertions of the leg adductors were pain-free, as was the lumbar spine.

The stretch status of the leg muscles was consistently good (quadriceps, hamstrings, leg adductors), but the hip flexor was slightly shortened and strongly toned on both sides. No anatomical or functional leg length discrepancy was detectable. The sacroiliac joints were not restricted in movement.

### 2.2. Imaging Procedures

**Figure 1:**
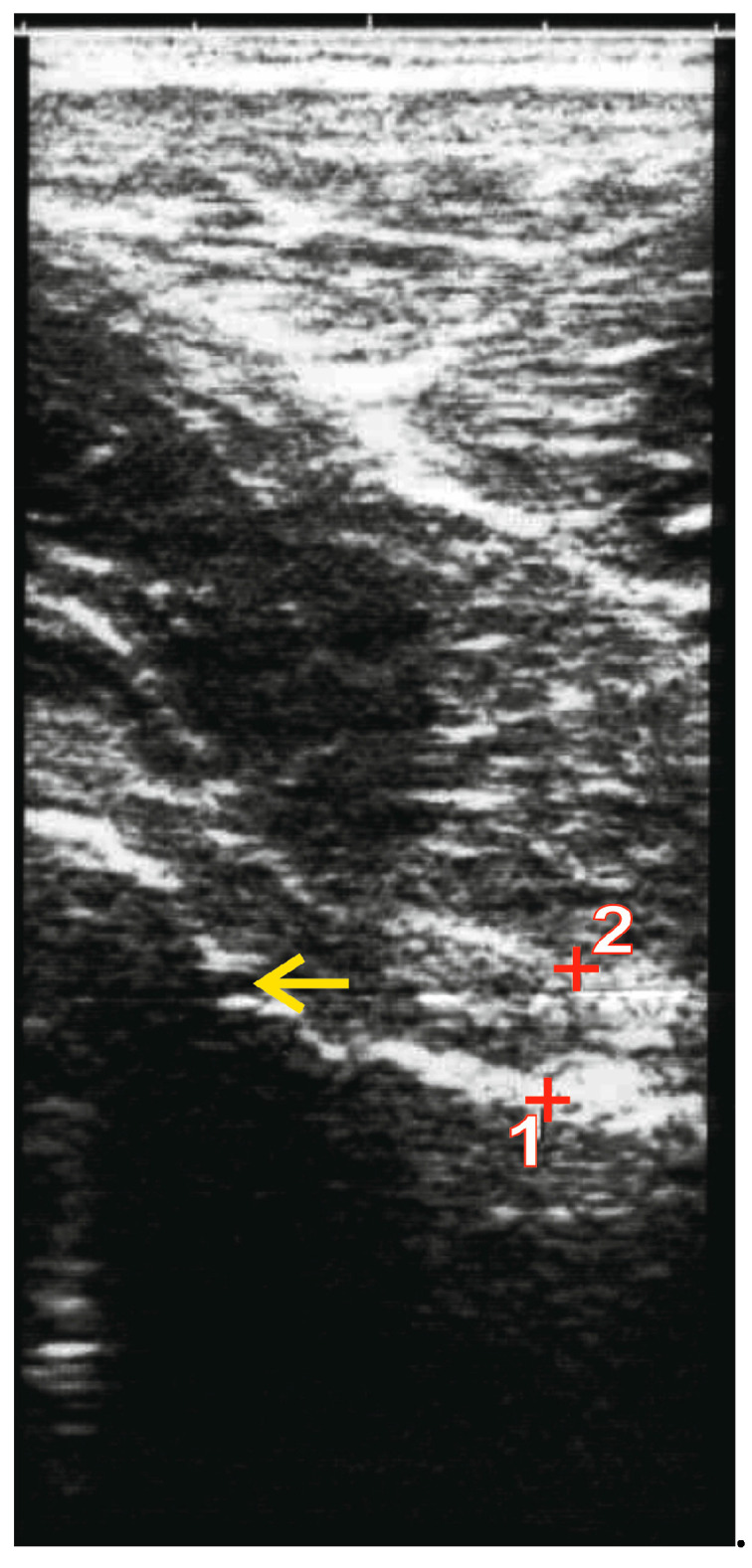
Ventral longitudinal section (parallel to femoral neck). Irregularity of cortical bone probably in the area of former epiphyseal joint (arrow) with disharmonious contouring of femoral head–neck connection. Distance between cortical bone of femoral neck (marker 1) and joint capsule (marker 2) of 5.8 mm is within normal range, with no intra-articular increase in volume. Ultrasound image was contrast-enhanced.

An ultrasound image of the hip with the patient in a supine position with hip rotation (hip and knee extension, big toe at 12 h) shows, in the ventral longitudinal section parallel to the femoral neck, an irregularity at the femoral head–neck junction (Figure 1), which appeared to be localized at the former epiphyseal joint, and which could be interpreted as incipient cam impingement.

**Figure 2:**
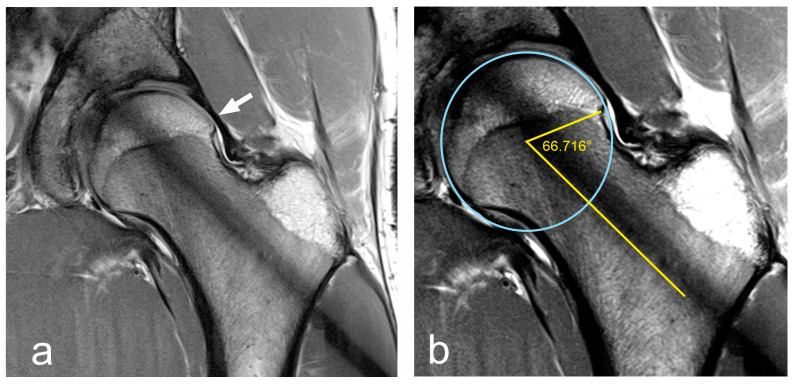
(**a**) Radial proton-dense sequence showing typical cam deformity (arrow). (**b**) Offset reduction due to the cam morphology with an alpha angle of 67°. Technical note: the representation in (**b**) was graphically enhanced to better visualize the reference lines.

As a high-resolution 3T MRI was available for the examination, we deviated from the usual diagnostic protocols (e.g., [15]) for medical ethical reasons (radiation exposure) and took MRI images directly (Siemens Magnetom Vida, Siemens Healthcare GmbH, Eschborn, Germany) 3 Tesla field strength, proton density sequence, TR 2000 ms, TE 35 ms, slice thickness 3 mm, radial acquisition in 30° steps adapted to the femoral neck; Figure 2a). This additionally had the advantage of being able to detect nonosseous or soft cam lesions [16]. One generally accepted assessment criterion in X-ray or MRI images is the alpha angle [17]. The reference range differs slightly in the literature. For example, Agricola et al. propose a threshold of 60° to define the presence of a cam deformity [18], meanwhile Barrientos et al. define an alpha angle of 57° as the diagnostic cut-off value [19] and Bisciotti et al. propose 55° [15]. In this case, an alpha angle of 67.8° was measured, which clearly indicated a CAM-FAI (Figure 2b).

Griffin et al. propose a triad of symptoms, signs and radiological features for the diagnosis of hip impingement [20]. All three of the diagnostic criteria proposed by the Warwick consensus statement [20] were met, as follows:

Symptoms:

- Exertional pain.
- Stiffness of the joint after exercise.

Clinical signs:

- Positive FADIR test.
- Apprehension test slightly painful.
- Restricted hip ROM.

Imaging findings:

- Irregularity at the femoral head–neck junction.
- Alpha angle > 60°.

### 2.3. Biomechanical Examinations

As the athlete had already undergone regular biomechanical examinations at the youth academy for several years, comparisons could be made with earlier tests. For each test, 3D posture scans (Balance 4D, Paromed, Neubeuern, Germany), 2D posture measurements (Dartfish Pro Suite 6, Dartfish, Fribourg, Switzerland) and isometric maximum strength tests (Backcheck 607, Dr. Wolff GmbH, Arnsberg, Germany) were carried out. Three-dimensional stereophotogrammetry is a well-proven measurement technique whose reliability and validity have been confirmed [21,22]. The 3D scanner used utilizes a Vialux scanning unit (Vialux GmbH, Chemnitz, Germany) which is medically approved and whose accuracy and reliability have been proven in previous studies [23]. The standardized 2D posture measurements carried out are based on videography. The accuracy and reliability of this method have been intensively investigated and fulfil all scientific quality criteria [24,25].

**Figure 3:**
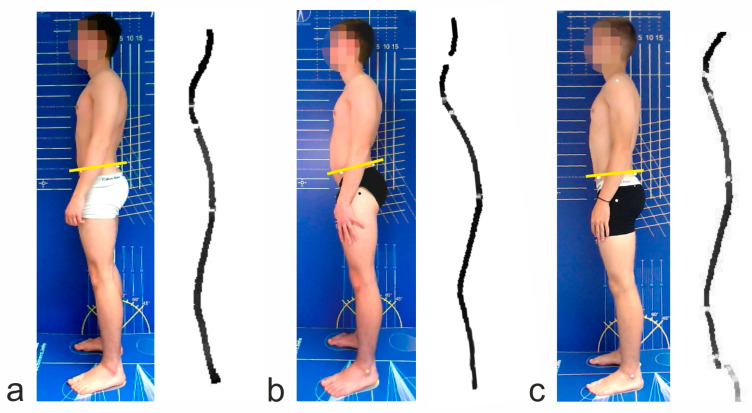
Posture in sagittal plane with pelvic incidence angle drawn and 3D reconstruction of lateral back profile. Marker spheres on ASIS and PSIS define the pelvic incidence angle (yellow bar). (**a**) Baseline measurement 6 months before complaints; (**b**) measurement at time of complaints; (**c**) after 8 weeks of therapy.

The postural analysis showed that, compared to the baseline measurements 6 months before the onset of pain (Figure 3a), the patient had developed a significant hyperlordosis of the lumbar spine with an increased forward tilt of the pelvis of 15° (Figure 3b). At the same time, a decrease in maximum strength of the abdominal muscles (trunk flexors) was measurable (Table 2, compare columns 1, 2 and 3). When asked, the athlete stated that he had changed his strength training 3 months prior and had increasingly trained the chest and leg area (m. pectoralis, m. quadriceps femoris) as well as the arm muscles (m. biceps, triceps brachii). This was confirmed in the strength measurements, which showed a significant increase in the strength of the trunk extensors, the pectoralis muscle and the quadriceps femoris muscle in the range of 12 to 17%.

**Figure 4:**
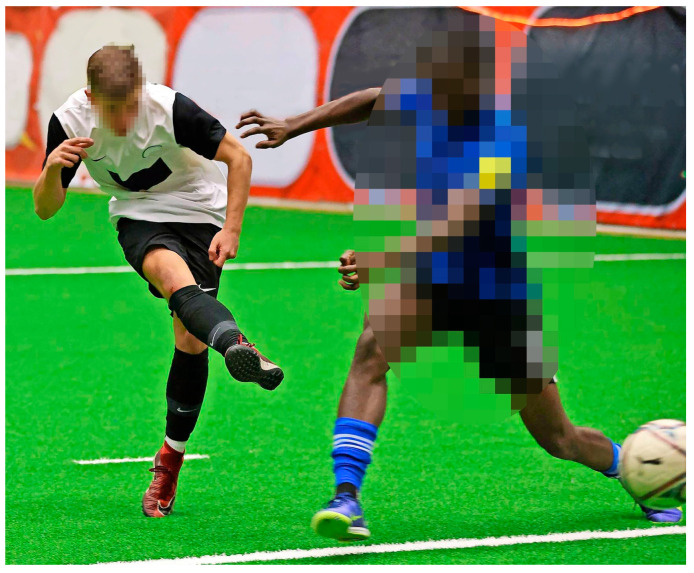
Example of a typical shooting position of examined athlete (left): shooting leg (right leg; movement on left is analogous) is flexed at hip joint, externally rotated and adducted, and upper body is strongly tilted forward and rotated.

During his training and matches, it was observed that the football player displayed strikingly strong hip flexion and adduction with twisting of the upper body and kicking leg during shots. This movement pattern was observed when shooting with both the right and left foot. A photo taken during an indoor tournament shortly before the onset of the symptoms shows this movement as an example (Figure 4).

### 2.4. Biomechanical Considerations

**Figure 5:**
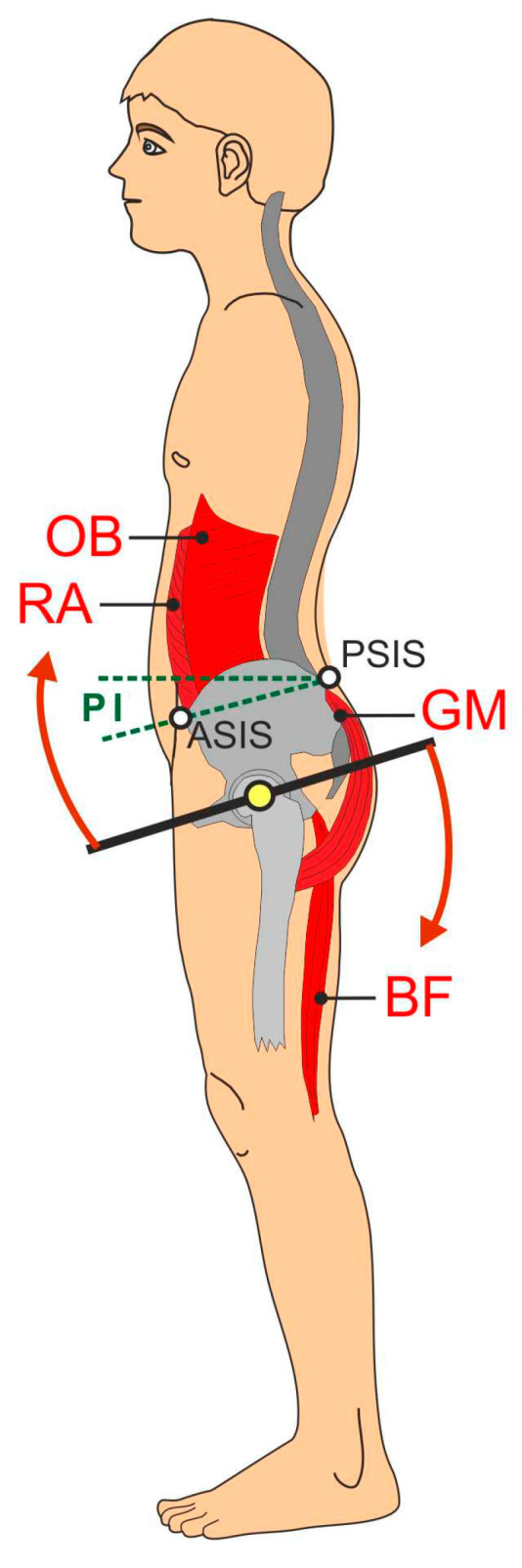
Muscular balance of pelvis in sagittal plane. RA, rectus abdominis; OB, obliquus; GM, gluteus maximus; BF, biceps femoris; PSIS, posterior superior iliac spine; ASIS, anterior superior iliac spine; PI, pelvic incidence angle. Arrows indicate rotation of pelvis around femoral head in terms of reduced pelvic incidence angle.

It is well known that weakness of the abdominal muscles, and especially of the gluteal muscles, causes the pelvis to tilt forward [26,27]. This is favoured by too strong or shortened lumbar back extensors, a too strong rectus femoris muscle, and a toned iliopsoas muscle (Figure 5).

We assumed that in this case, the improperly trained musculature with a simultaneously toned, poorly stretched hip flexor was the cause of the increased pelvic forward tilt as a result of a muscular imbalance.

The following biomechanical considerations should help to understand our approach to avoid unfavourable contact between the acetabulum and femoral head during kicking in the context of CAM-FAI by correcting the pelvic resting position.

**Figure 6:**
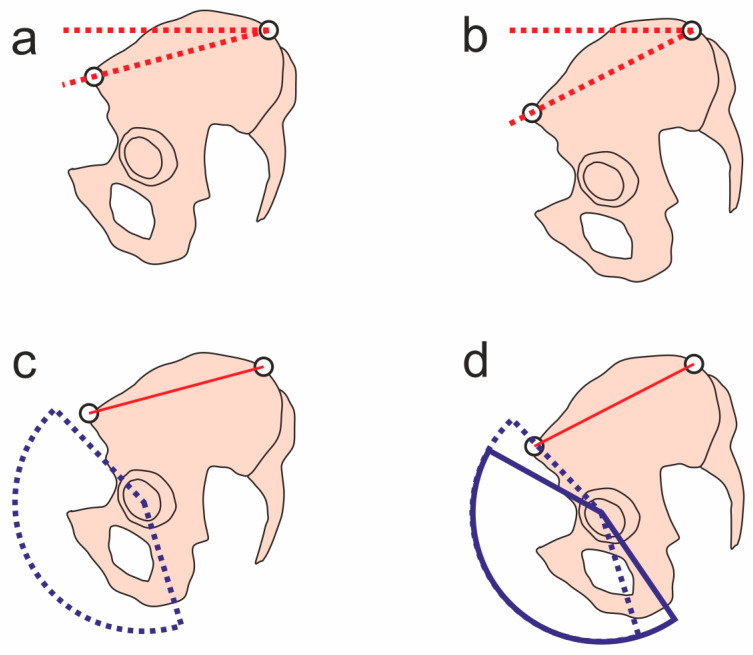
(**a**) Pelvis in sagittal plane in neutral position; white dots mark ASIS and PSIS; red lines mark the pelvic incidence angle (**b**) Pelvis with increased forward tilt (pelvic incidence angle). (**c**) ROM of femur with pelvis in neutral position (dotted blue line). (**d**) Displacement of ROM with pelvis in forward tilt (solid blue line). Modified after [29].

**Figure 7:**
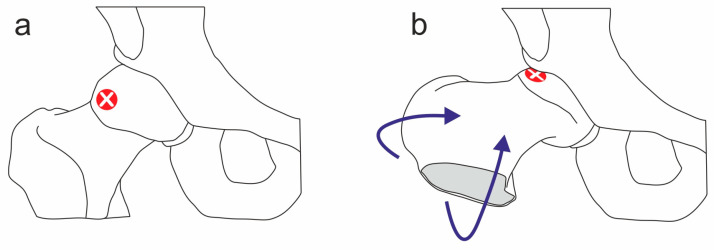
Biomechanical consideration: collision of cam impingement (red dot) with acetabulum ((**a**)—neutral position) during combined flexion, adduction and internal rotation (**b**). Based on [7].

An increased (anteverted) pelvic tilt already changes the position of the femoral neck in relation to the acetabulum in the resting position (Figure 6). If the leg is now flexed and internally rotated during a shot, from a biomechanical point of view, earlier contact between the ventral acetabulum and the femoral head is favoured, which will be additionally enhanced by an existing cam impingement (Figure 7).

From a biomechanical point of view, strong flexion in the hip joint during the kicking movement is often caused by kicking muscles that are too weak (hip flexors). According to the principle of the optimal acceleration path and according to Hochmuth [28], the athlete must cover a greater acceleration trajectory in order to be able to transfer the same impulse to the ball with reduced force (due to weak musculature). In the case of a shot, this would be an initially stronger dorsal backswing of the shooting leg and/or increased flexion in the hip joint, both of which lengthen the circular path covered by the shooting foot and thus increase the transmitted impulse. Increased trunk flexion in the lumbar region towards the end of hip flexion can raise the pelvis ventrally (reduced pelvic tilt), thus additionally increasing the total range of motion. In this case, such strong hip flexion with rotation and flexion of the entire upper body could be observed during the shot (Figure 4).

### 2.5. Therapy

According to these biomechanical considerations, the following therapy goals were chosen:

1. Strengthening of the hip extensors (m. gluteus maximus, hamstrings) to reduce the pelvic tilt.
2. Strengthening of the abdominal muscles/trunk flexors (m. rectus abdominis, m. obliquus) to reduce the pelvic tilt.
3. Strengthening and detoning of the hip flexors (m. iliopsoas) to reduce maximum hip flexion during kicking.

During a 2-week break from football, the athlete worked on strengthening the above muscle groups 3 times a week. For this purpose, hypertrophy training was performed twice a week on machines, 3–4 sets with 10–12 repetitions each and a 1 min set break (Table 3). Additionally, whole-body electromyostimulation (Miha Bodytec 2 WB-EMS, Miha Bodytec, Augsburg, Germany) training was performed once a week (impulse width 350 µs, bipolar impulse without impulse increase, 4 s load and 4 s break intervals, overall training duration 20 min), with a focus on strengthening the gluteal, abdominal, hamstring, adductor and abductor muscles.

In parallel, he performed stretching and mobilization exercises for the hip joint in the pain-free area twice a week.

## 3. Results and Outcome

After 2 weeks, the athlete took part in football training again, initially with reduced intensity (light passing exercises, no duels, no abrupt changes of direction). The groin pain occurred less frequently (pain scale 5 out of 10). During this time, muscle training and the therapeutic intervention were maintained.

After another 2 weeks, the athlete resumed regular football training, but initially without playing in league matches. Muscle training and therapy continued as before. The player reported infrequent groin pain rated 3–4 out of 10.

Eight weeks after the start of the first intervention, the player was able to resume participation in training and games without any complaints. A follow-up examination showed a significant reduction in the pelvic inclination angle (Table 3) and a return to normal lumbar lordosis (Figure 3c). The success of strength training was verified by increased muscle strength (Table 3). Even after a further six months, the player was still pain-free despite the now more intensive training and match load.

## 4. Discussion

In this case study, a CAM-FAI was diagnosed using the criteria defined by [20]. In their review, Peters et al. concluded that there is a great inconsistency regarding the indications for the surgical treatment of CAM-FAI [30]. In addition to pain lasting longer than 6 months, the most frequently mentioned surgical criteria include an alpha angle > 50°, reduced ROM for hip flexion and internal rotation and a positive impingement sign. Most of these criteria were met in this case study. Nevertheless, any surgical intervention should be preceded by an attempt at conservative therapy.

In the therapy commonly used to date, the hip adductors and/or abductors in particular are trained on a patient-specific basis, the lower trunk strength is improved and stabilization exercises are performed [11].

However, the therapeutic approach in this case study was different. It is known that in certain hip joint positions (flexion/internal rotation), CAM-FAI leads to faulty contact between the femoral head and the acetabulum or labrum [14]. Based on the observation that the athlete’s pelvic position in the sagittal plane had deteriorated significantly in recent months, the idea was to bring his pelvic position from an increased forward tilt back to a neutral position. At 15°, the pelvic tilt measured at the time of the complaints was clearly outside the reference range (Table 2). For example, ref. [31] found a mean pelvic tilt of 9.4° ± 4.1° in men (95% confidence interval: 8.6–10.2°) as a reference range. After the end of our treatment, a forward tilt of 6° was again achieved within the normal range.

With a regular pelvic position, a maximal regular 130° hip flexion (pelvic coordinate system) leads to lifting of the thigh to 20° above the horizontal level (world coordinate system) [7]. If the pelvis tilts forward, for example, due to a muscular imbalance [27], the final position of the leg in the world coordinate system is clearly closer to the horizontal level (Figure 6). If the flexion capacity is reduced at the same time due to cam impingement, the more unfavourable pelvic position promotes the occurrence of pain [32]. When shooting with simultaneous adduction and internal rotation of the leg, impingement is biomechanically favoured (Figure 7) [33].

Footballers, in particular, are susceptible to the occurrence of unilateral imbalances caused by the different requirement profiles of the kicking leg and supporting leg [34]. Such imbalances can also lead to asymmetries in the pelvic region and are therefore often associated with complaints [35,36]. In our case study, however, the imbalances were found in the dorso-ventral relation. An increased pelvic tilt (measured by the angle between ASIS and PSIS to the horizontal level) is usually caused by a muscular imbalance. The muscle groups that lift the anterior part of the pelvis (gluteus maximus, rectus abdominis, hamstrings) are often too weak and the pelvic-lowering muscles (rectus femoris, iliopsoas) are too strong or hypertonic [27]. The latter two muscles are usually very developed in football players because they are used for hip flexion (the rectus femoris muscle is also responsible for knee extension) during kicking [37]. In this case study, hip flexion muscles that were too weak may have led to increased hip and trunk flexion in shooting situations. Unbalanced strength training combined with a lack of stretching and flexibility exercises had obviously created an imbalance between the pelvic lifting muscles and the pelvic lowering muscles and led to an increased pelvic tilt. The combination of the mentioned biomechanical and muscular factors probably caused earlier unfavourable contact between the acetabulum and the femoral head and thus caused pain during the shooting movement. This is in line with the study by Patel et al., who suggested an improvement of the pelvic tilt to minimize faulty contacts in the hip joint caused by impingement [10]. Therefore, our therapeutic approach consisted of a symmetrical treatment protocol with the aim of improving the global position of the pelvis.

In this case study, targeted muscle training improved the pelvic tilt angle, which ultimately led to freedom from pain in the presence of cam impingement. This confirmed the simulation-based assumptions of Kobayashi et al. [9].

Our conclusions are limited by a number of points. Even though repositioning the pelvis caused the symptoms to disappear and a biomechanical cause is plausible, we cannot prove a causal relationship. As hip pain can be caused by several entities together [1,15], our study only shows one possible therapeutic approach. Furthermore, this therapeutic approach is limited to athletes with an excessive pelvic tilt.

Therefore, future studies should investigate the relationship between the pelvic tilt and complaints in CAM-FAI with a larger number of cases.

## 5. Conclusions

A change in the pelvic position in terms of an increased pelvic tilt due to a muscular imbalance caused by non-adapted strength training can lead to pain in the groin region in the presence of unfavourable anatomical conditions (disturbance of the femoral head–neck junction in terms of CAM-FAI) and unfavourable movement sequences (shooting technique with increased hip and trunk flexion), even if there is no sport-related increase in load. Therefore, it is important to examine the statics of the pelvic, in particular the pelvic tilt, in the case of suddenly occurring groin complaints. Knowledge of the pelvic position in the context of medical diagnostics is important in order to be able to use conservative therapy in a targeted manner to correct existing postural deficits. Possible inappropriate strength training performed by the athlete should be kept in focus.

## Figures and Tables

**Table 1:** Movement measurements of hip joints according to neutral zero (°).

Movement	Left	Right
Extension/flexion	10–0–125	10–0–130
Abduction/adduction	40–0–30	40–0–30
External/internal rotation (hip joints in 90° flexion)	45–0–20	45–0–35

**Table 2:** Posture and force parameters at different time points.

	T(−1)	Delta T(−1) → T(0)	T(0)	Delta T(0) → T(+1)	T(+1)
Pelvic tilt (°)	9°	+6°	15°	−9°	6°
Trunk flexors (N)	715	−4.9%	680	+21.3%	825
Trunk extensors (N)	760	+12.5%	855	+0.6%	860
Chest muscles (N)	568	+15.7%	657	+0.5%	660
Quadriceps (N)	980	+17.3%	1150	−1,7%	1130
Hamstrings (N)	430	+2.8%	442	+25.3%	554
Ham/quad ratio (–)	0.44		0.38		0.49

Isometric maximum forces in newtons. Delta indicates changes between 2 measurement times. T(−1) = 6 months before pain occurred. T(0) = examination time. T(+1) = 8 weeks after beginning of therapy.

**Table 3:** Exercise intervention.

Muscles	Exercises	Sets	Repetitions
Gluteus maximus	Hip thrust	3	12
	Plank, one leg extended	3 each side	10
Rectus abdominis	Sit-ups on exercise ball	3	12
Hamstrings	Nordic hamstrings	4	5
Iliopsoas	Leg lift in prone position	4	10
	Stretching in prone position	3 each side	40 s

## Data Availability

The data presented in this study are available on request from the corresponding author. The data are not publicly available to protect the privacy of the patient.
